# Incorporation and Conduction of Protons in Ca, Sr, Ba-Doped BaLaInO_4_ with Ruddlesden-Popper Structure

**DOI:** 10.3390/ma12101668

**Published:** 2019-05-22

**Authors:** Nataliia Tarasova, Irina Animitsa, Anzhelika Galisheva, Daniil Korona

**Affiliations:** Institute of Natural Sciences and Mathematics, Ural Federal University, 620000 Yekaterinburg, Russia; irina.animitsa@urfu.ru (I.A.); jelya95@gmail.com (A.G.); D.V.Korona@urfu.ru (D.K.)

**Keywords:** Ruddlesden-Popper structure, oxygen-ion conductivity, proton conductivity, water uptake

## Abstract

The new phases BaLa_0.9_M_0.1_InO_3.95_ (M = Ca^2+^, Sr^2+^, Ba^2+^) with a Ruddlesden-Popper structure were obtained. It was established that all investigated samples were capable for the water uptake from the gas phase. The ability of water incorporation was due to not only by the presence of oxygen vacancies, but also due to the presence of La-O blocks in the structure. The degree of hydration of the samples was much higher than the concentration of oxygen vacancies and the composition of the samples appear to be BaLaInO_3.42_(OH)_1.16_, BaLa_0.9_Ca_0.1_InO_3.25_(OH)_1.4_, BaLa_0.9_Sr_0.1_InO_3.03_(OH)_1.84_, BaLa_0.9_Ba_0.1_InO_2.9_(OH)_2.1_. The degree of hydration increased with an increase in the size of the dopant, i.e., with an increase in the size of the salt blocks. It was proven that doping led to the increase in the oxygen ionic conductivity. The conductivities for doped samples BaLa_0.9_M_0.1_InO_3.95_ were higher than for undoped composition BaLaInO_4_ at ~1.5 order of magnitude. The increase in the conductivity was mainly attributed to the increase of the carrier concentration as a result of the formation of oxygen vacancies during doping. The proton conductivities of doped samples increased in the order Ca^2+^–Sr^2+^–Ba^2+^ due to an increase in the concentration of protons. It was established that all doped samples demonstrated the dominant proton transport below 450 °C.

## 1. Introduction

Protonic ceramics are important materials for using as electrolytes in proton-conducting solid oxide fuel cells (H-SOFCs) [1,2,3,4,5,6]. The application of the proton-conducting complex oxides in comparison with oxygen-ion conductors (in oxygen ion-conducting solid oxide fuel cells O-SOFCs) has several advantages. First, the proton electrolytes have lower activation energies of charge carriers (~0.5 eV) than the oxygen-ion conductors (~1 eV). Consequently, they have higher conductivities. This allows us to decrease the operating temperatures from 900–1000 °C (O-SOFCs) to 500–700 °C (H-SOFCs) [7] and provides the decrease of the production cost of H-SOFCs at 27–37% [8]. Secondly, the water vapor in the H-SOFCs is produced at the air-side electrode (the cathode side) and this avoids the dilution of fuel by steam [9], and, consequently, increases the energy conversion efficiency [10,11]. Therefore, the discovery of a new structure family of proton conductors is important both in terms of the development of scientific materials and in the applied aspect.

The most studied protonic conductors are the complex oxides with perovskite or perovskite-related structures. Further development of proton conductors suggests investigations of materials with new types of structures, including Ruddlesden-Popper structure. It is common understanding that oxygen-deficient compounds are capable of manifesting not only oxygen-ionic conductivity, but also protonic conductivity in a humid atmosphere. Therefore, perovskite-related structures with oxygen-ion conductivity can be tested as potentially capable of proton transport. Recently, researchers uncovered new data about oxygen-ion transport in the complex oxides with a Ruddlesden-Popper structure [12,13,14]. The phase BaNdInO_4_ has a structure that is slightly different from K_2_NiF_4_ [15]. The BaNdInO_4_-type structure (monoclinic, P2_1_/c) consists of alternative stacking of the [NdO] oxide unit and Nd_1/4_Ba_3/4_InO_3_ perovskite unit with the InO_6_ edge-facing Nd−O units.

The new compounds BaYInO_4_, BaSmInO_4_, BaHoInO_4_, BaErInO_4_, and BaYbInO_4_ were also synthesized [16,17], but the total electrical conductivity of BaNdInO_4_ was the highest among BaRInO_4_ compounds. Therefore, the investigations were mainly focused on BaNdInO_4_. The phase BaNdInO_4_ was described as a mixed oxygen ionic-electronic conductor [12,13,14] and cation doping with different metals M = Ca, Sr, Ba, Ce, Ga, Cr, Si, Mg, Zr, Nb, Ta, Ti, and Sn at the Nd-sites or In-sites were used for increasing the oxide ion conductivity [13,18]. It was shown that oxygen-ion conductivity of Ca^2+^ and Ti^4+^ doped compositions was higher than those for other dopants [13,18]. It was proven that the increase of oxygen-ion conductivity at acceptor doping is due to the formation of oxygen vacancies. At the same time, the possibility of the formation of the interstitial oxygens in the rock-salt block of BaNdIn_1-*x*_Ti*_x_*O_4+δ_ due to donor doping, which leads to the increase of oxide-ion conductivity, was also considered [18]. The interstitial oxygen conduction was also discussed in the donor doped K_2_NiF_4_-type LaSrInO_4_ oxides [19,20]. It should also be mentioned that Kotaro Fujii et al. [17] emphasized that there is no direct evidence for the interstitial oxygen. In the BaNdInO_4_-type structure, there is no rock-salt block, but the A rare earth oxide unit could have an influence on the impurity phases. All this demonstrates that these phases are very interesting and further investigation is required.

Although oxygen-ion transport in the systems based on BaNdInO_4_ was widely discussed, no studies of proton transport have been performed. Nevertheless, the possibility of proton transport for phases with the Ruddlesden-Popper structure was described earlier for oxyfluorides Ba_2_InO_3_F and Ba_3_In_2_O_5_F_2_ [21]. K_2_NiF_4_-type materials of Pr_1-*x*_M_1+*x*_InO_4_ (M = Ba^2+^, Sr^2+^; *x* = 0, 0.1) exhibit proton conductivity and are suitable for the operation of proton-conducting solid oxide fuel cells (H-SOFCs) at targeted temperatures of 500 to 700 °C [22]. The layered perovskites Sr_1+*x*_Sm_1−*x*_AlO_4−δ_ and Sr_1+*x*_Pr_1−*x*_AlO_4-δ_ also have proton conductivity and high proton transport number at 700 to 900 °C [23]. These studies also show that, for a correct discussion, the conductivity measurements for phases with a layered structure should be carried out in atmospheres with controlled humidity.

To develop the novel proton conductors, we focused on the composition of BaLaInO_4_ as a parent compound, which has a Ruddlesden-Popper structure. The structural features of the phases of the composition A^II^LnInO_4_ (AA′BO_4_) were described earlier [24], where the relationship between transitions of K_2_NiF_4_−CaFe_2_O_4_ type structures was established. The compound BaLaInO_4_ crystallizes in an orthorhombic structure with space group *Pbca* and with lattice parameters *a* = 12.933 (3), *b* = 5.911 (1), and *c* = 5.905 (1) Å [24]. In the present work, the new complex oxides BaLa_0.9_M_0.1_InO_3.95_ (M = Ca^2+^, Sr^2+^, Ba^2+^) with a Ruddlesden-Popper structure were obtained via the solid-state method. The structure, water uptake, and proton transport were examined. The influence of the nature of dopant (alkaline earth metals) on the transport properties were discussed.

## 2. Materials and Methods

The phases BaLa_0.9_M_0.1_InO_3.95_ (M = Ca^2+^, Sr^2+^, Ba^2+^) and the parent composition BaLaInO_4_ were prepared by a solid-state method. The preliminary dried high-purity powders of CaCO_3_, SrCO_3,_ BaCO_3_, In_2_O_3_, and La_2_O_3_ were used. Prior to weighing, the starting materials were dried. The stoichiometric powder mixtures were manually milling in an agate mortar and then calcined at 800, 900, 1000, 1100, 1200, and 1300 °С for 24 h. Intermediate grindings were made for every following heating step.

Phase purity was monitored by powder X-ray diffraction (XRD) on a Bruker Advance D8 diffractometer with CuK*_α_* radiation. The crystal structures of dry and hydrated samples were determined by Rietveld refinement [25]. The dry samples were prepared for XRD by heat treated at 1100 °C for 4 h and then cooled in dry Ar (*p*H_2_O = 3.5 × 10^−5^ atm). The hydrated samples were obtained at slow cooling from 1100 to 150 °C (1 °C/min) under a flow of wet Ar (99.999% purity,* p*H_2_O = 2 × 10^−2^ atm). Ar atmosphere was used to avoid any carbonization of the samples.

TG-analysis was carried out on a STA 409 PC analyzer (Netzsch, Selb, Germany) coupled with a quadrupole mass spectrometer QMS 403 C Aëolos (Netzsch, Selb, Germany) in order to determine the degree of hydration. The samples were heated at the rate of 10 °C/min in a corundum crucible under a flow of dry Ar at temperatures ranging from 40 to 1100 °C. Before TG-measurements, the powder samples were initially hydrated as described above. The number of moles of water per one formula unit was determined and the notation of the total composition as BaLa_0.9_M_0.1_InO_3.95_·*n*H_2_O was used for convenience of comparison. To indicate that H_2_O incorporation gives OH^−^-groups, the formulas were also written in another representation as BaLa_0.9_M_0.1_InO_3.95−*n*_(OH)_2*n*_.

The ceramics used for the electrical measurements were prepared by pressing cylindrical pellets and sintering them at 1300 °C for 24 h in dry air. The samples typically had a relative density of around ∼92% (density of the sintered samples was determined by the Archimede method). After polishing, the platinum paste electrodes were fired at 900 °C for 3 h. The *ac* conductivity of the samples (two-probe method) was measured over the frequency range of 1 kHz–1 MHz using a Z-1000P (Elins, RF) impedance spectrometer at an applied voltage of 150 mV. The least squares refinement program ZView (Scribner Associates Inc., Southern Pines, NC, USA) was used to fit the impedance data to an (RQ) equivalent circuit, where R is resistance and Q is the constant phase element. The bulk resistance was calculated from the impedance data and then the bulk conductivity was calculated and discussed below. From the fitted resistances and, together with the geometrical dimensions, the total conductivity can be calculated using the following equation.

(1) $$\sigma =\frac{1}{R}\cdot \frac{l}{S}$$

where *l* and *S* are the thickness and surface area of the sample.

The measurements of the temperature dependencies of conductivities were performed in the temperature range from 200 to 1000 °C every 10 to 20 °C with a cooling rate of 1 °C/min. During the measurement, the sample was held at each temperature until the resistances became constant. The conductivity measurements were carried out under dry and wet air or Ar.

The «wet» gas was obtained by bubbling the gas at room temperature first through distilled water and then through a saturated solution of KBr (*p*H_2_O = 2 × 10^−2^ atm). The «dry» gas was produced by circulating the gas through P_2_O_5_ (*p*H_2_O = 3.5 × 10^−5^ atm). The humidity of gases was measured by the H_2_O-sensor (“Honeywell” HIH-3610, Charlotte, NC, USA).

## 3. Results and Discussions

### 3.1. X-ray Characterization

The X-ray diffraction (XRD) results for the parent compound BaLaInO_4_ and its doped analogs BaLa_0.9_M_0.1_InO_3.95_ (M = Ca^2+^, Sr^2+^, Ba^2+^) showed that the samples were single phase (orthorhombic symmetry, space group *Pbca*). The obtained lattice parameters for the sample BaLaInO_4_ were in good agreement with previously reported data [24]. The substitution of M^2+^ ions for La^+3^ ion led to changes in structural parameters (Table 1). The lattice parameters and cell volumes increased with an increasing ionic radius of the dopant [26]. It should be noted that a more significant increase in parameters and cell volume in comparison with the undoped sample was observed for the Ba^2+^-doped sample. At the same time, for the Ca^2+^-doped sample (as the closest-sized ion to lanthanum), an increase and decrease in the lattice parameters was observed. It is known that the formation of oxygen vacancy is also accompanied by decreases of the lattice parameter because of the size relationship of oxygen vacancy and the oxide ion [27]. We can assume that, for the Ca^2+^-doped sample, the overall effect of the influence of the size of the B-cation and oxygen vacancy occurred and no monotonic change of the parameters was observed.

The hydration processes led to the changes in the crystal structure for the samples compared with anhydrous samples. All hydrated samples BaLaInO_4_∙*n*H_2_O and BaLa_0.9_M_0.1_InO_3.95_∙*n*H_2_O belonged to the monoclinic symmetry (space group *P*2/*m*). The increase in the ionic radii of alkaline earth metal led to the increase in the cell volume of hydrate samples (Table 1).

**Figure 1:**
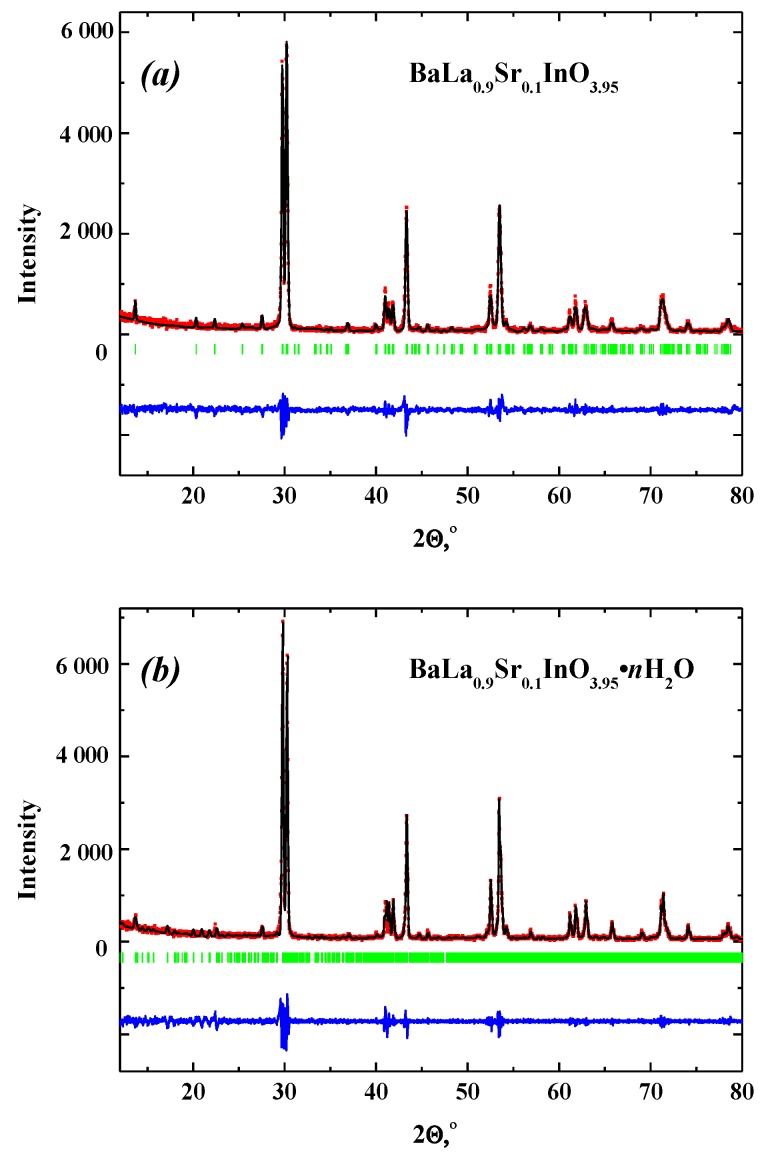
XRD patterns of BaLa_0.9_Sr_0.1_InO_3.95_ (**a**) and BaLa_0.9_Sr_0.1_InO_3.95_∙*n*H_2_O (**b**). At the bottom of the figure, the pattern is the difference between the experimental (red) and the calculated one (dark) after refinement. Vertical bars show the Bragg angle positions.

It should be noted that XRD-patterns for all obtained samples were refined by the Rietveld analysis. Results for anhydrous BaLa_0.9_Sr_0.1_InO_3.95_ and hydrated BaLa_0.9_Sr_0.1_InO_3.95_∙*n*H_2_O are presented in Figure 1a,b as an example of fitting.

### 3.2. Thermal Properties

The water uptake was established by TG-analysis in combination with differential scanning calorimetry (DSC) and mass-spectrometry (MS).

**Figure 2:**
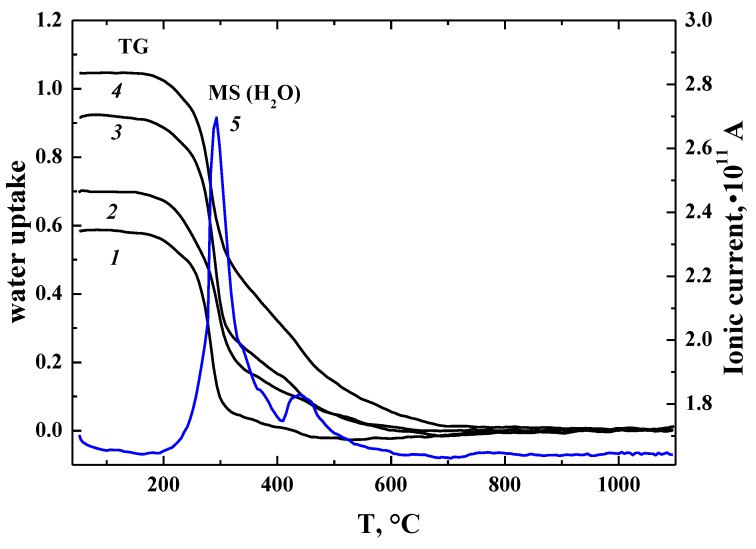
TG data for hydrated samples BaLaInO_4_∙*n*H_2_O (*1*), BaLa_0.9_Ca_0.1_InO_3.95_∙*n*H_2_O (*2*), BaLa_0.9_Sr_0.1_InO_3.95_∙*n*H_2_O (*3*), BaLa_0.9_Ba_0.1_InO_3.95_∙*n*H_2_O (*4*), and MS (H_2_O)-data for BaLa_0.9_Ba_0.1_InO_3.95_∙*n*H_2_O (*5*).

As is well known, the possibility of water uptake from the gas phase for perovskite-type oxide conductors is due to the presence of oxygen vacancies in the structure and can be described as follows.

(2) $$V_{o}^{••}+H_{2}O+O_{o}^{\times }\Leftrightarrow 2{\left(OH\right)}_{o}^{•}$$

where $V_{o}^{••}$ is the oxygen vacancy, $O_{o}^{\times }$ is the oxygen atom in a regular position, and ${\left(OH\right)}_{o}^{•}$ is the hydroxyl group in the oxygen sublattice. Based on the concentration of oxygen vacancies of obtained samples BaLaInO_4_[V_O_]_0_ and BaLa_0.9_M_0.1_InO_3.95_[V_O_]_0.05_, there should be a degree of hydration of 0 and 0.05 mol H_2_O per formula unit. As shown in Figure 2, for all the investigated samples, the mass loss was observed, which is attributed to a loss of water, in accordance with MS data. Water uptake of the samples was much higher than the concentration of oxygen vacancies and the composition of the samples appears to be BaLaInO_4_∙0.58H_2_O (or BaLaInO_3.42_(OH)_1.16_*,* which represents that H_2_O incorporation gives OH^−^-groups, BaLa_0.9_Ca_0.1_InO_3.95_∙0.7H_2_O (or BaLa_0.9_Ca_0.1_InO_3.25_(OH)_1.4_), BaLa_0.9_Sr_0.1_InO_3.95_∙0.92H_2_O (or BaLa_0.9_Sr_0.1_InO_3.03_(OH)_1.84_), and BaLa_0.9_Ba_0.1_InO_3.95_∙1.05H_2_O (or BaLa_0.9_Ba_0.1_InO_2.9_(OH)_2.1_).

**Figure 3:**
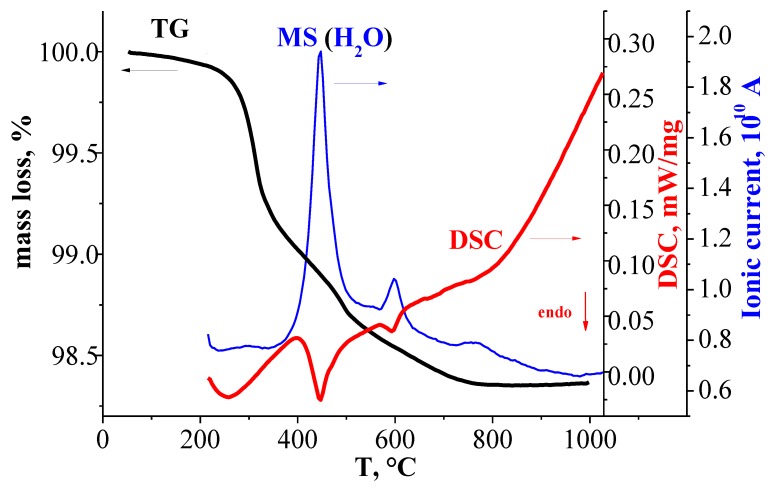
TG-(dark line), DSC-(blue line), and MS (H_2_O)-data (red line) for hydrated sample BaLaInO_4_∙*n*H_2_O.

Figure 3 represents the comparison of TG-curves, DSC-curves, and MS-curves for the hydrated sample BaLaInO_4_∙*n*H_2_O. As can be seen, the sample underwent a mass decrease in the temperature from 200 to 800 °C, which was accompanied by a three endothermic DSC-signals (two well pronounced and one weak signal) at ~320 °C, ~500 °C, and ~700 °C. The MS analysis indicated that the mass change can be attributed by the water loss. No other volatile components (CO_2_, O_2_) were detected. The doped samples BaLa_0.9_M_0.1_InO_3.95_ showed the similar TG-curves, DSC-curves, and MS-curves (Figure 2).

It is known that the ability of water incorporation is ensured not only by the presence of oxygen vacancies, but also by the features of the crystal structure [28]. For layered structures, it can be explained by the ability of water incorporation by the salt La−O blocks, which alternates with blocks of the perovskite matrix. For example, the formation of hydrated phases based on the Ruddlesden-Popper-type layered oxide Ba_2_ZrO_4_ [29] was proven. During hydration, this phase can incorporate up to 1.7 mol of water Ba_2_ZrO_4_∙1.7H_2_O. The effects of water incorporation into layered structures were summarized in the dissertation [30]. It was shown that RP phases can adopt up to two water molecules per formula unit/rock-salt block. At the same time, the large amount of water molecules in some RP-phases can be explained by the formation of hydrates and, in addition, not all RP compounds react with water under ambient conditions. This shows that the elemental composition of an RP compounds has a significant effect on the stability, and atoms with the biggest influence are the cations present in the rock-salt block. If the majority of cations in the rock-salt block are large cations, e.g., Ba, K, Rb or Cs, then the compounds have a higher tendency for the intercalation of water into the rock-salt block [30].

As can be seen from Figure 2, the degree of hydration increases in the order of BaLaInO_4_–BaLa_0.9_Ca_0.1_InO_3.95_–BaLa_0.9_Sr_0.1_InO_3.95_–BaLa_0.9_Ba_0.1_InO_3.95_. We can suggest that, with an increase in the size of the dopant, the size of the salt block increases. This contributes to the placement of a larger number of OH^−^-groups, which occupy interstitial sites within the rock salt layers.

Therefore, we can conclude that investigated samples BaLaInO_4_ and BaLa_0.9_M_0.1_InO_3.95_ can show the ability for water uptake not only in accordance with reaction (2), but also due to the presence in the structure of La-O blocks (rock-salt blocks).

(3) $$BaLaInO_{4}+\frac{x}{2}H_{2}O\to BaLaInO_{4-\frac{x}{2}}{\left(OH\right)}_{x}$$

(4) $$BaLa_{0.9}M_{0.1}InO_{3.95}+\frac{x}{2}H_{2}O\to BaLa_{0.9}M_{0.1}InO_{3.95-\frac{x}{2}}{\left(OH\right)}_{x}$$

Thus, the results show the ready ability of the RP-structure to incorporate extra water as OH^−^-groups, located in the sites of the rock salt layers. This ability can lead to proton transport. In addition, it is important to note that these results also show that it is possible to regulate the concentration of intercalated water by the nature of the dopant introduced into the salt layers.

### 3.3. Electrical Properties

**Figure 4:**
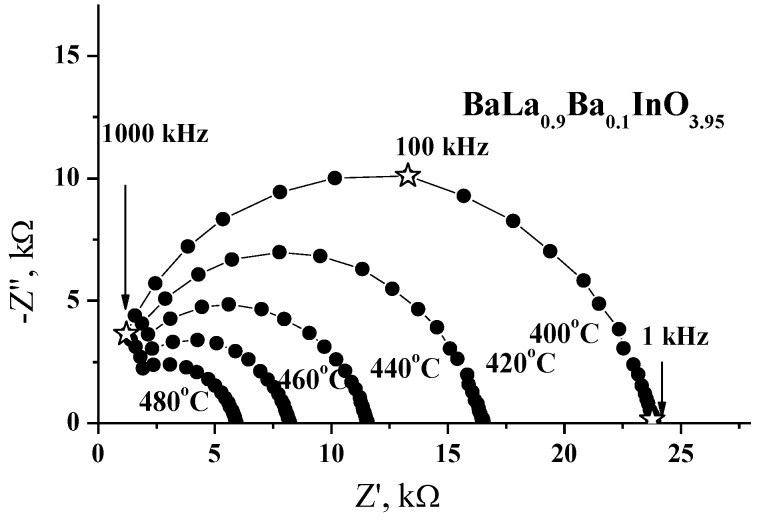
Evolution of the impedance spectra with temperature for the sample BaLa_0.9_Ba_0.1_InO_3.95_ in dry air (*p*H_2_O = 3.5 × 10^−5^ atm).

For all the samples in this study, one semicircle, starting from zero coordinates of the complex Z-Z′ plane and corresponding to the bulk component, was observed. Typical impedance spectroscopy diagrams for the BaLa_0.9_Sr_0.1_InO_3.95_ sample, recorded at various temperatures, are given in Figure 4. The capacitance of the observed arc was estimated to be about 10^−11^ F∙cm^−1^, which is typical of a bulk response. According to the experimental data obtained for other phases with an RP structure (for example, LaSrInO_4_ [19,20] and NdBaInO_4_ [13]), two contributions can be observed in the typical impedance spectroscopy diagrams. The semicircle in the high-frequency range, corresponding to the contribution from bulk resistance, and very small low-frequency semicircle, attributed to the contribution of grain-boundary resistance. It should be noted that the grain-boundary arc was manifested in the frequency range of 100 mHz to 100 Hz. In comparison with the presented data for doped BaLaInO_4_ (Figure 4), the observed one semicircle is realized in the frequency range of 1 to 1000 kHz. Therefore, the arc corresponding to the grain boundary response, which appears at lower frequencies (in comparison with bulk semicircle), cannot be observed due to the instrumental limitations of the frequency range.

### 3.4. Dry Conditions

**Figure 5:**
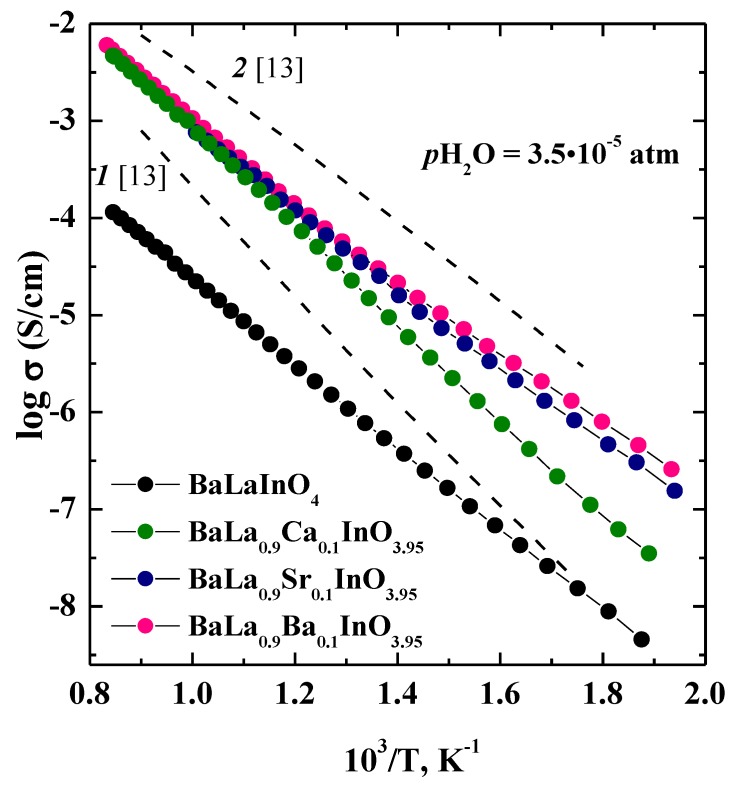
The temperature dependencies of the total conductivities (*p*O_2_ = 0.21 atm) of BaLaInO_4_ and BaLa_0.9_M_0.1_InO_3.95_ (M = Ca^2+^, Sr^2+^, and Ba^2+^) in dry air and comparison with (*1*) BaNdInO_4_ and (*2*) Ba_1.1_Nd_0.9_InO_3.95_ in air.

**Figure 6:**
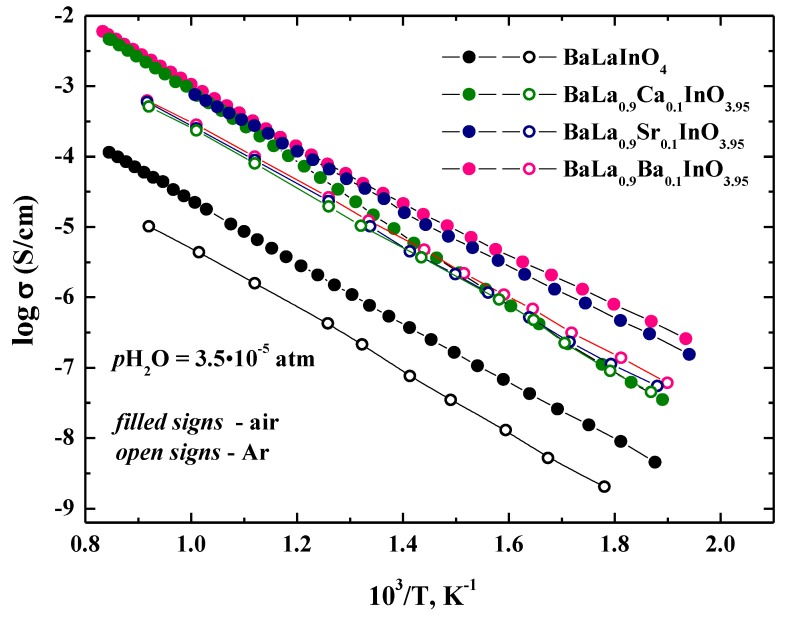
The temperature dependencies of the conductivities of BaLaInO_4_ and BaLa_0.9_M_0.1_InO_3.95_ (M = Ca^2+^, Sr^2+^, and Ba^2+^) in dry air (filled signs) and dry Ar (open signs).

The temperature dependencies of total conductivities for investigated samples BaLaInO_4_ and BaLa_0.9_M_0.1_InO_3.95_ (M = Ca^2+^, Sr^2+^, Ba^2+^) are shown in Figure 5. For dry air (*p*H_2_O = 3.5 × 10^−5^ atm) (Figure 5), the conductivities for doped samples BaLa_0.9_M_0.1_InO_3.95_ are higher than those for undoped composition BaLaInO_4_ at a ~1.5 order of magnitude. To evaluate the contribution of electronic conductivity of investigated phases, the measurements were performed in the Ar atmosphere (that is, at lower *p*O_2_ where ionic conductivity dominates). The comparison of total conductivities in air and Ar under dry conditions was shown in Figure 6. The conductivities in Ar atmosphere were lower than the conductivities, measured in air. The conductivity enhancement in air can be ascribed to the electronic conduction because of formation of holes in air, as shown by the quasi-chemical reaction.

(5) Vo••+12O2⇔Oo×+2h•

For the undoped sample, the conductivity in Ar atmosphere were lower than the conductivities, measured in air, over the whole temperature range studied. This proves the mixed oxygen ionic-electronic character of conductivity of BaLaInO_4_. For Sr^2+^, Ba^2+^-doped samples, the conductivity exhibited the same character, but, for the Ca^2+^-doped sample, the differences were more significant at higher temperatures and the conductivity values were comparable at low temperatures, which indicates that the electron contribution decreases with a falling temperature and this phase exhibited the dominant oxygen–ionic character of conductivity below 500 °C. These results also showed that for the Sr^2+^, Ba^2+^-substituted phases, the contribution of the electronic conductivity was greater when compared to the Ca^2+^-substituted phase.

Thus, the observed increase in the ionic conductivity (Ar atmosphere) for doped phases BaLa_0.9_M_0.1_InO_3.95_ in comparison with undoped composition BaLaInO_4_ was mainly attributed to the increase of the carrier concentration as a result of the formation of oxygen vacancies during doping. The M^2+^-cations are incorporated into the lattice at La^3+^ sites with the creation of oxygen vacancies as charge-compensating defects. This doping process can be represented by the following defect reaction.

(6) $$2\mathrm{MO}\overset{La_{2}O_{3}}{\to }2M_{\mathrm{La}}^{\prime }+2O_{O}^{\times }+V_{O}^{••}$$

Comparing the ionic conductivities of the doped phases, we can note the tendency of its increase in the order of BaLa_0.9_Ca_0.1_InO_3.95_−BaLa_0.9_Sr_0.1_InO_3.95_−BaLa_0.9_Ba_0.1_InO_3.95_, i.e., in the order of increasing the ionic radius of dopants. The concentration of the oxygen vacancies was the same for the doped samples, so the difference in the conductivity of the BaLa_0.9_M_0.1_InO_3.95_ might be attributed to their mobilities. In this respect, effect of the dopant nature on the conductivity should be considered.

The authors [13] have investigated NdBaInO_4_, doped with Ca^2+^, Sr^2+^, Ba^2+^. Their results show another trend in comparison with the data obtained in this work: σ_ion_ of Ca^2+^ > Sr^2+^ > Ba^2+^. Their conductivities showed the tendency to increase with a decrease in the ionic radii of the M site ions, i.e., Ca^2+^ is the most favorable dopant on Nd^3+^ sites. The authors [13] indicate that the replacement of Nd^3+^ by the cations with the most comparable size may minimize local structural relaxation and this main reason may explain the enhancement of the oxygen vacancy conductivity in the order of Ca^2+^ > Sr^2+^ > Ba^2+^. Taking into account the differences in the radii of La^3+^ and Ca^2+^, Sr^2+^, and Ba^2+^, we could expect a similar trend in the change in conductivity of doped BaLaInO_4_. However, this is not the case. Clearly, other reasons should be considered.

There are not many reports in the literature on the relationships of O^2−^-transport in phases with the RP-structure. However, for the ABO_3_ perovskites, there is a lot of information on the influence of the nature of A-cations, B-cations, and dopants on the mobility of oxygen vacancies. In the general case, there are many factors that affect the mobility of oxygen vacancies. For phases with the same structure and with the same concentration of dopants, the main factors are generally identified.

(1) Geometric characteristics. It is well known that the size proportion of the A-cations and B-cations is an important factor for high ionic conductivity. The relationship of structure parameters and oxygen-ion transport properties had been studied by Wakamura [31]. The author plotted the activation energy (E_a_) versus lattice volume (V) for numerous ion conductors, and found a simple inverse correlation between them, according to the expression E_a_ = A_v_/V^2/3^, where A_v_ is a constant. The relationship indicates that the E_a_ value is decreased with a weakened long-range force due to the volume expansion. A similar tendency was observed for the ionic conductivity of mixed ionic-electronic conductors. For example, for Ln_0.5_M_0.5_FeO_3-δ_ (M = Sr^2+^, Ba^2+^) [32], increasing the mismatch between La^3+^ and M^2+^ radii weakens the metal-oxygen bonding and increases the oxygen mobility.

Returning to the analysis of the experimental data, presented in this paper, we can make the assumption concerning the reason for the enhancement of the oxygen vacancy conductivity in the order of Ca^2+^–Sr^2+^–Ba^2+^ for doped BaLaInO_4_. The observed increase in the lattice volume and the lattice parameters weakens the metal-oxygen bonding, and, as a consequence, increases the oxygen mobility. It should be noted that the activation energy for oxygen ion conduction in doped BaLaInO_4_ decreased in the order of Ca^2+^ (0.86 eV)–Sr^2+^ (0.85 eV)–Ba^2+^ (0.82 eV).

As can be seen, the results obtained for changes in oxygen-ion conductivity are consistent with the main trends described for perovskites. At the same time, it should be emphasized that the nature of the dopant has little effect on the oxygen ionic conductivity of doped BaLaInO_4_. The differences reached less than 0.3 order at low temperatures. This is due to the fact that the replacement of cations is carried out in the salt block and the change in geometrical parameters also occurs mainly in the salt block. The formation of oxygen vacancies and their migration occurs in the perovskite block. Therefore, the nature of the dopant introduced into the salt block does not significantly affect the oxygen ionic conductivity.

(2) Binding energies for dopant−oxygen vacancy pair clusters. It is well-known that interactions between dopant ions and their charge-compensating defects can lead to the formation of clusters, which can trap the migrating species. This implies that the oxide ion vacancies might be strongly bound to the dopant species, and the migration of the oxygen vacancy might depend on the binding energy of the dopant-oxygen vacancy, which, consequently, leads to lower oxide ion conductivities. As for the calculations for dopant-vacancy clusters in Ca^2+^-, Sr^2+^-, and Ba^2+^-doped NdBaInO_4_ with RP-structure, it was shown [13] that the calculated binding energies are comparable to ca.–0.9 eV, which indicates that trapping for the migrating oxygen vacancies in NdBaInO_4_ from the Ca^2+^, Sr^2+^, and Ba^2+^ dopants could be comparable. If we assume that the same tendency is observed for the doped phase BaLaInO_4_, then it can also be said that the reason for the cluster formation does not explain the change in conductivity due to the nature of the dopant.

This result shows that the nature of the dopant, by occupying sites within the rock salt layers, does not have a very significant effect on the oxygen-ion conduction and that the key parameter governing the oxygen-ion conductivity of these materials is the concentration of oxygen vacancies. Likely, the variation in the concentration of oxygen vacancies of solid solutions, which will be studied in the future, will reveal new regularities of ionic transport in phases with the RP-structure.

The comparison of the conductivities of the investigated samples based on BaLaInO_4_ with Nd-structural analogues BaNdInO_4_ [13] showed the total and ionic conductivities were in good agreement and comparable for Sr^2+^ and Ba^2+^ doped phases at low temperatures, but Nd-structural analogues exhibited higher conductivities at higher temperatures [13]. Figure 5 shows a comparison of the temperature dependencies of conductivity for undoped phases BaLaInO_4_ and BaNdInO_4_ and Ba^2+^ doped phases BaLa_0.9_Ba_0.1_InO_3.95_ (or Ba_1.1_La_0.9_InO_3.95_), Ba_1.1_Nd_0.9_InO_3.95_ [13].

### 3.5. Wet Conditions

**Figure 7:**
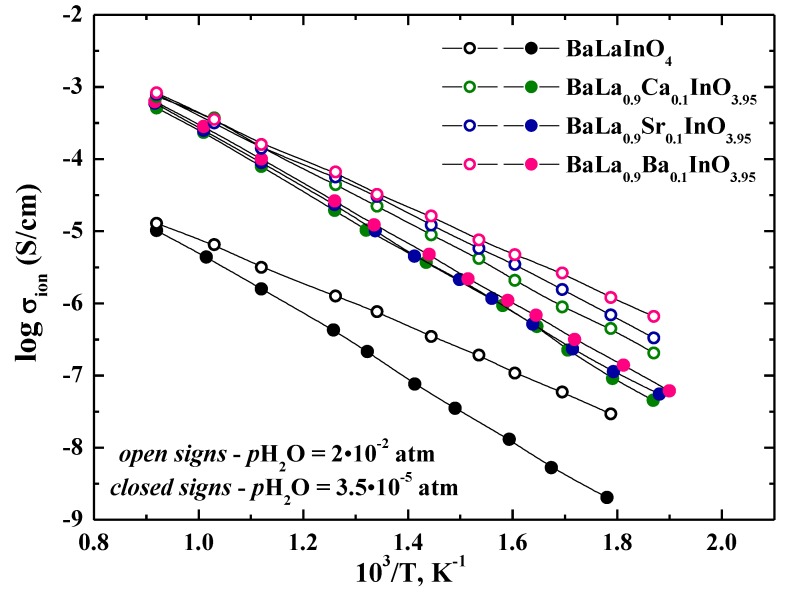
The temperature dependencies of the ionic conductivities of BaLaInO_4_ and BaLa_0.9_M_0.1_InO_3.95_ (M = Ca^2+^, Sr^2+^, Ba^2+^) at dry Ar (filled signs) and wet Ar (closed signs).

Figure 7 shows a comparison of the temperature dependencies of conductivity of investigated samples in Ar under dry and wet conditions. For wet Ar (*p*H_2_O = 2 × 10^−2^ atm) (Figure 7, curves with open signs), the conductivities of all samples were higher than those in dry Ar. Clearly, this behavior was a result of the insertion of water and the formation of proton charge carriers. The conductivity differences were more significant at lower temperatures as the hydration increased with decreasing temperature. We can suppose that the nature of dopant does not have significant effects on the type of dominant charge carrier, and increasing in the conductivity values in wet atmospheres is due to the appearance of proton carriers for all investigated samples.

**Figure 8:**
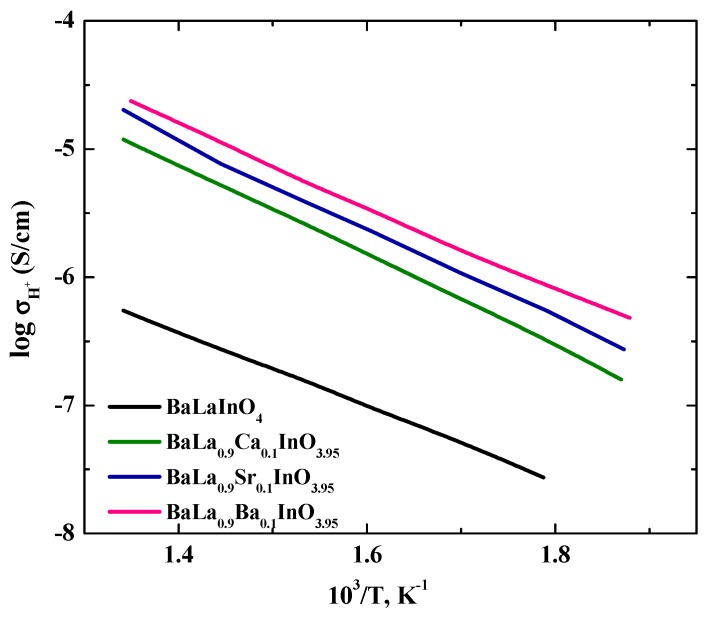
The temperature dependencies of the proton conductivities of BaLaInO_4_ and BaLa_0.9_M_0.1_InO_3.95_ (M = Ca^2+^, Sr^2+^, and Ba^2+^).

The proton conductivity was calculated as the difference between the conductivity in wet and dry Ar, that is, σ_H_ = σ (wet Ar) − σ (dry Ar), and the temperature dependencies are shown in Figure 8.

As can be seen from Figure 8, the proton conductivities of doped samples increased in the order Ca^2+^–Sr^2+^–Ba^2+^. We can assume that these differences were due to an increase in the concentration of protons in the same order. The activation energies decreased in the order of Ca^2+^ (0.70 eV)–Sr^2+^ (0.68 eV)–Ba^2+^ (0.65 eV). These values are greater than the activation energy of proton transport in doped perovskites ~0.5 eV [33]. As was discussed above in the analysis of TG data, during hydration, the main part of the protons was in the salt block. This situation is like hydroxides. It is well known that proton mobility is dependent on the strength of hydrogen bonds with the next-nearest oxygen atoms and energy barriers are high for proton transport in non-H-bonded systems [34,35]. It is clear that proton migration in the layer of salt block is a difficult in comparison with the proton migration in the bulk of perovskite block. Moreover, from a general point of view, four key steps for hydration can be distinguished [36]: these are (1) water adsorption on the oxide surface, (2) proton migration from the surface to the bulk of the oxide, (3) proton migration in the bulk, and (4) oxide ion vacancy migration in the bulk. The proton migration from the surface to the bulk is difficult. For investigated systems, the proton migration from the salt layer is assumed to be the same as the proton migration from the surface to the bulk of the perovskite structure. Taking into account these differences in the structures of perovskites and phases with the RP-structure, as well as differences in hydration mechanisms, it can be concluded that the proton transport in the investigated phases is realized with higher activation energies compared to the substituted ordinary perovskites.

**Figure 9:**
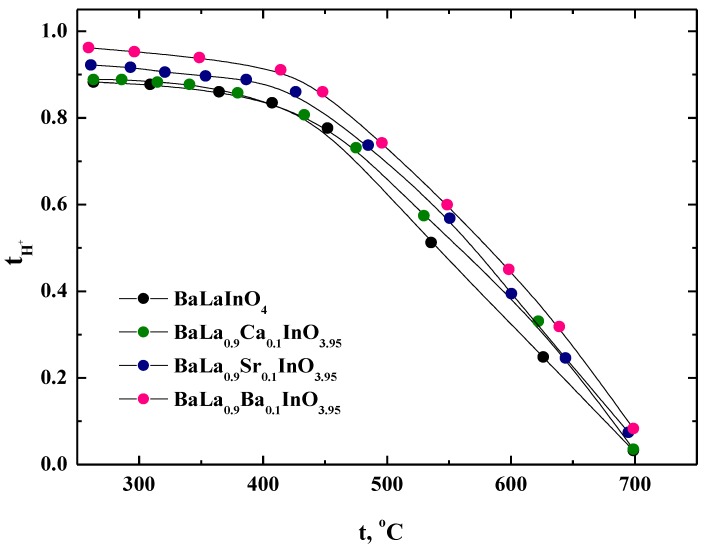
The temperature dependencies of the proton transport numbers of BaLaInO_4_ and BaLa_0.9_M_0.1_InO_3.95_ (M = Ca^2+^, Sr^2+^, and Ba^2+^).

The proton transport numbers were calculated according to the formula t_H_ = σ_H_/σ (wet air) and were plotted as a function of temperatures in Figure 9. It can be seen that the proton transport numbers increased with decreasing temperature and the investigated samples were nearly pure proton conductors below 450 °C.

The results indicate that new phases BaLa_0.9_M_0.1_InO_3.95_ (M = Ca^2+^, Sr^2+^, Ba^2+^) might be a promising proton-conducting oxide in future applications. Further modification of the composition will improve the H^+^-conductivity.

## 4. Conclusions

The new phases BaLa_0.9_M_0.1_InO_3.95_ (M = Ca^2+^, Sr^2+^, and Ba^2+^) with the Ruddlesden-Popper structure were obtained. The analysis of the effect of nature of dopant (alkaline earth metals) on the hydration processes and on the transport properties of BaLaInO_4_ was carried out. It was established that all investigated samples were capable of water uptake from the gas phase. The ability of water incorporation was due to, not only the presence of oxygen vacancies, but also due to the presence in the structure of La-O blocks. The degree of hydration increased with a growth in the size of the dopant, i.e., with an increase in the size of the salt blocks, that contributed to the placement of a larger number of OH^−^-groups, which occupied interstitial sites within the rock salt layers.

It was proven that doping led to the increase of the oxygen ionic conductivity values. The conductivity values for doped samples BaLa_0.9_M_0.1_InO_3.95_ were higher than for undoped composition BaLaInO_4_ at a ~1.5 order of magnitude. The increase in the conductivity values was mainly attributed to the increase of the carrier concentration as a result of the formation of oxygen vacancies during doping. The oxygen ionic conductivity of doped samples increased in the order of BaLa_0.9_Ca_0.1_InO_3.95_–BaLa_0.9_Sr_0.1_InO_3.95_–BaLa_0.9_Ba_0.1_InO_3.95,_ i.e., in the order of increasing the ionic radius of dopants. The increase in the lattice volume and the lattice parameters weakens the metal-oxygen bonding, and, as a consequence, increases the oxygen mobility. The activation energy for oxygen ion conduction decreased in the same order.

The conductivity increased in wet conditions as a result of the insertion of water and the formation of proton charge carriers. The proton conductivity was estimated by subtracting the conductivity data for dry Ar from the corresponding data in wet Ar. The proton conductivities of doped samples increased in the order Ca^2+^–Sr^2+^–Ba^2+^ due to an increase in the concentration of protons. The activation energies for the proton conductivities were 0.70–0.68–0.65 eV, respectively.

## Figures and Tables

**Table 1:** Cell parameters and volume of anhydrous and hydrated samples.

Sample	*a*, Å	*b*, Å	*c*, Å	β, ^o^	Cell Volume, (Å^3^)
BaLaInO_4_	12.932(3)	5.906(0)	5.894(2)	90	450.188(2)
BaLa_0.9_Ca_0.1_InO_3.95_	12.967(4)	5.913(3)	5.884(5)	90	451.224(2)
BaLa_0.9_Sr_0.1_InO_3.95_	12.950(2)	5.917(1)	5.897(5)	90	451.911(4)
BaLa_0.9_Ba_0.1_InO_3.95_	13.002(1)	5.919(3)	5.901(3)	90	454.183(7)
BaLaInO_4_∙*n*H_2_O	12.717(4)	14.763(4)	7.214(9)	92.92(6)	1352(8)
BaLa_0.9_Ca_0.1_InO_3.95_∙*n*H_2_O	12.710(8)	14.770(8)	7.218(6)	92.82(2)	1353(6)
BaLa_0.9_Sr_0.1_InO_3.95_∙*n*H_2_O	12.725(3)	14.791(2)	7.220(4)	92.79(0)	1357(4)
BaLa_0.9_Ba_0.1_InO_3.95_∙*n*H_2_O	12.741(0)	14.806(5)	7.222(6)	92.75(2)	1360(9)
